# The genesis and evolution of the African Field Epidemiology Network

**Published:** 2011-12-14

**Authors:** David Mukanga, Mufuta Tshimanga, Frederick Wurapa, Fred Binka, David Serwada, William Bazeyo, George Pariyo, Fred Wabwire-Mangen, Sheba Gitta, Stella Chungong, Murray Trostle, Peter Nsubuga

**Affiliations:** 1 African Field Epidemiology Network, Kampala, Uganda; 2 Zimbabwe Field Epidemiology Training Program, Department of Community Medicine, University of Zimbabwe, Harare, Zimbabwe; 3 School of Public Health, University of Ghana, Accra, Ghana; 4 School of Public Health, Makerere University, Kampala, Uganda; 5 World Health Organization, Geneva; 6 United States Agency for International Development, Washington DC, USA; 7 Center for Global Health, Centers for Disease Control and Prevention, Atlanta, USA

**Keywords:** African Field Epidemiology Network, AFENET, Network

## Abstract

In an effort to contain the frequently devastating epidemics in sub-Saharan Africa, the World Health Organization (WHO) Regional Office for Africa launched the Integrated Disease Surveillance and Response (IDSR) strategy in an effort to strengthen surveillance and response. However, 36 sub-Saharan African countries have been described as experiencing a human resource crisis by the WHO. Given this human resource situation, the challenge remains for these countries to achieve, among others, the health-related Millennium Development Goals (MDGs). This paper describes the process through which the African Field Epidemiology Network (AFENET) was developed, as well as how AFENET has contributed to addressing the public health workforce crisis, and the development of human resource capacity to implement IDSR in Africa. AFENET was established between 2005 and 2006 as a network of Field Epidemiology Training Programs (FETPs) and Field Epidemiology and Laboratory Training Programs (FELTPs) in Africa. This resulted from an expressed need to develop a network that would advocate for the unique needs of African FETPs and FELTPs, provide service to its membership, and through which programs could develop joint projects to address the public health needs of their countries. A total of eight new programs have been developed in sub-Saharan Africa since 2006. Programs established after 2006 represent over 70% of current FETP and FELTP enrolment in Africa. In addition to growth in membership and programs, AFENET has recorded significant growth in external partnerships. Beginning with USAID, CDC and WHO in 2004-2006, a total of at least 26 partners have been added by 2011. Drawing from lessons learnt, AFENET is now a resource that can be relied upon to expand public health capacity in Africa in an efficient and practical manner. National, regional and global health actors can leverage it to meet health-related targets at all levels. The AFENET story is one that continues to be driven by a clearly recognized need within Africa to develop a network that would serve public health systems development, looking beyond the founders, and using the existing capacity of the founders and partners to help other countries build capacity for IDSR and the International Health Regulations (IHR, 2005).

## Introduction

In 2006, the World Health Organization (WHO) indicated that there was a global shortfall of 4.3 million trained health workers, which was most severe in 57 countries, 36 of which are in sub-Saharan Africa. The health-related Millennium Development Goals (MDGs) cannot be not be achieved in Africa without an adequate health workforce [1]. Investments made through global health initiatives, private foundations, multi- and bi-lateral development agencies have had a diminished impact because of a lack of human and institutional capacity to absorb, deploy and use their funds efficiently [2].

In 1993, the World Health Organization's Regional Office for Africa (WHO/AFRO) at its annual Regional Committee (which is the governance body for the organization) meeting in Gaborone, Botswana, called on its Member States to strengthen their public health surveillance systems to address the frequently devastating epidemics that the region was experiencing [3]. Following the Gaborone meeting, and in an effort to strengthen surveillance and response to infectious diseases, WHO/AFRO developed the Integrated Disease Surveillance and Response (IDSR) strategy in 1998 [4–5]. IDSR was endorsed and supported by several partners including the United States (U.S.) Agency for International Development (USAID) and the U.S. Centers of Disease Control and Prevention (CDC). In 2001, as part of their support for IDSR, these partners started investing in applied public health training in several African countries to develop a public health workforce that could implement IDSR with an initial focus on strengthening outbreak investigation and response and related surveillance activities [5].

One of the proven strategies for building public health surveillance and response systems and the workforce to operate said systems is implementation of Field Epidemiology and Laboratory Training Programs (FELTPs) [6–8]. FELTPs build and strengthen public health systems, while simultaneously training future public health leaders. Because FELTP trainees provide service during training, FELTPs create a setting in which evidence-based public health systems that serve communities effectively and efficiently can be established [9]. Without strong field epidemiology capacity, African countries will be unable to build and use disease surveillance and response systems, and remain vulnerable to disease threats [10].

Field Epidemiology Training Programs (FETPs) were established in Africa to address the critical shortage of epidemiological skills within the public health workforce [6,9,11]. The first FETPs in Africa were established in the early 1990s as partnerships between the ministries of health (MOH), universities, district local governments, and other partners as part of the Rockefeller Foundation supported Public Health Schools Without Walls (PHSWOW) project [8,9,12–13]. These programs shared experiences, training curricula, learning materials, and staff, and also undertook joint field epidemiology projects. All PHSWOWs, FETPs, and FELTPs are members of the global FETP network, the Training Programs in Epidemiology and Public Health Interventions Network (TEPHINET) which was established in the late 1990s. TEPHINET addresses the needs of FETPs around the world including curriculum and accreditation issues.

The need for a regional applied epidemiology training network in Africa was recognized in the late 1990s because of the peculiarities in the African region [12]. For instance, most programs in Africa tended to have more active involvement of universities or other training institutions compared to FETPs elsewhere because of the need for formally-recognized university qualifications by the graduates for career progression. African programs also tended to include health services management as part of their curriculum to address the needs of decentralization of health services from national to sub-national levels.

## The Genesis of AFENET

Two meetings were held in 2004 and 2005 in Uganda and Ghana respectively, to develop a vision, mission, and strategy for an African field epidemiology network. The meetings were attended by Program Directors and representatives of MOHs from each of the four existing African TEPHINET member programs at the time (Ghana (a 1-year PHSWOW), Kenya (an FELTP) [14], Uganda (a 2-year PHSWOW), and Zimbabwe (a 2-year PHSWOW)), and partners from WHO, CDC and USAID.

At the 2004 meeting, participants noted the following needs:1)To strengthen and standardize field epidemiology curricula and training strategies across programs and train critical program staff; 2) To define critical issues for sustainability that including expanding the pool of partners supporting the programs both technically and financially; 3) To add a laboratory component into the training programs to strengthen public health laboratory practice for effective surveillance and response; 4) To share experiences and help other countries in Africa to develop their own FETPs and FELTPs, or be linked into existing ones to address their training needs; 5) To broaden the scope of African FETPs and FELTPs to address other public health beyond infectious diseases; 6) Membership by programs in global networks was not sufficient to support the unique situation of the African continent.

Meeting participants also recognized that global disease initiatives to address polio, malaria, tuberculosis (TB) and HIV/AIDS (e.g., the Global Fund Against TB, AIDS, and Malaria (GFATM)) were using the few existing local public health experts to implement their vertical programs but had not yet contributing significantly to developing new public health experts.

Participants at the meeting agreed that it was imperative to develop a regional network that would address these issues including finding ways to encourage global initiatives, other donors and governments to include funding for strengthening of field epidemiology capacity in Africa in a concerted manner.

**Role of partners in the process:** After the partners agreed to form AFENET they worked collaboratively to develop ideas into proposals for submission to potential funding partners to expand the FETPs and FELTPs on the continent, as well as establish a secretariat of the new organization to coordinate, mainstream, and advocate for the programs. One such example was a jointly developed proposal to address health systems strengthening which was endorsed by Country Coordination Mechanisms (CCMs) in Ghana, Uganda, and Zimbabwe and submitted to the GFATM. CCMs are the GFATM′s tool to promote local ownership and participatory decision-making through their diverse membership that characteristically comprises representatives from both public and private sectors, including governments, multilateral or bilateral agencies, non-governmental organizations, academic institutions, private businesses and people living with the diseases. Although the proposal was never funded, it helped build a strong bond between the programs. The concerted effort as highlighted by the CCM endorsements demonstrated the value of the programs to their countries, and the commitment in host countries to invest in these programs.

Each partner used its unique capabilities to develop AFENET. The MOHs and universities brought their leadership, goodwill, and time commitments, and they agreed to establish AFENET as a network and mechanism for promoting and sustaining the programs. The universities, within which the programs were anchored, signed a Memorandum of Understanding (MOU) to establish AFENET and agreed to collaborate through AFENET to advance field epidemiology capacity within their institutions, countries, and the continent. The MOHs in the founder countries agreed to let the universities sign the MOU which outlined how AFENET resources would be managed within the countries. In each country the AFENET resources were to be managed jointly by the MOH and the university. The universities and MOHs agreed to develop and use AFENET as the principal mechanism for field epidemiology training development in Africa.

WHO was involved in the development of the vision for the organization, and provided technical support especially from the Lyon office during the early development of AFENET. USAID and CDC provided technical input into the organizational strategy, and provided seed funding to establish the AFENET secretariat, as part of the existing mechanisms for outbreak investigation training. USAID, through a contracted organization, supported the development of the governance and leadership framework [15]. In addition to initial technical and financial investments, CDC utilized AFENET as its primary implementing partner for FELTP development in Africa.

## Evolution of AFENET

**Figure 1:**
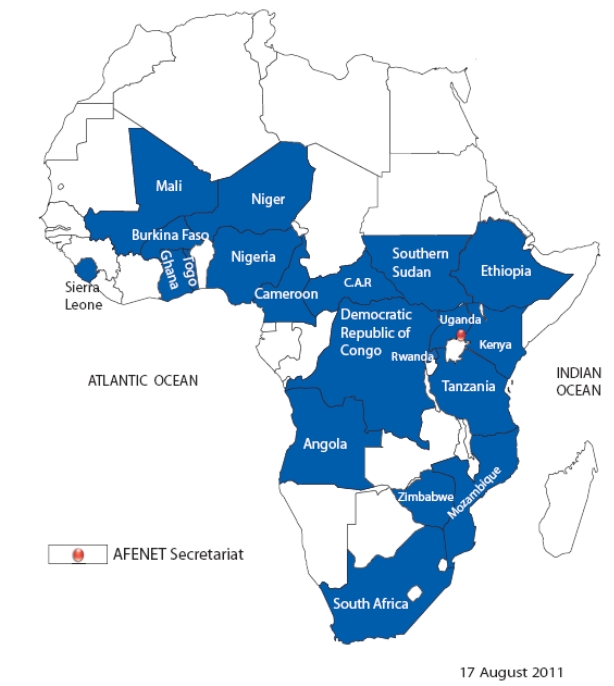
African Field Epidemiology Network (AFENET) member countries

Since the creation of AFENET, a total of eight new programs have been developed [8]. Since some of the programs serve more than one country (regional FELTPs) another eight countries have since become affiliated with AFENET. The AFENET constitution describes the membership of AFENET, and the process to achieve membership (by countries and institutions) [16]. AFENET has evolved in its scope, not only networking and supporting FETPs and FELTPs, but developing and implementing public health projects with its membership. Figure 1 shows the countries that currently constitute the AFENET Network. Over the last 5 years, AFENET has recorded internal growth from four to 20 countries from West, East, and Southern Africa and covering a large part of sub-Saharan Africa.

### Organization

**Figure 2:**
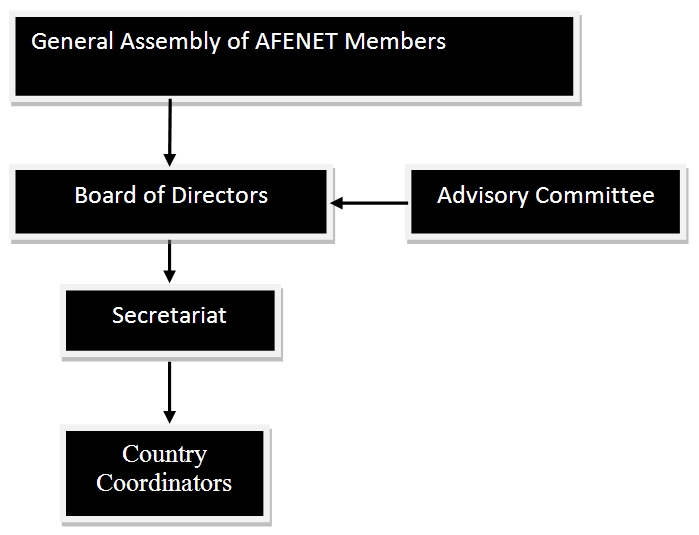
African Field Epidemiology Network (AFENET) governance Structure

Figure 2 describes the governance structure of AFENET. The highest decision making organ of AFENET is its general assembly, which is made up of all the network's members and meets once a year. Every 2 years the general assembly elects the Board of Directors[16]. The Board of Directors is responsible for formulation of policies to govern the operations of AFENET, and oversees the promotion and realization of the vision, mission and objectives of AFENET. The board is advised by an advisory committee consisting of eminent public health experts from around the continent who are invited as-needed. The secretariat coordinates and manages the day to-day affairs of the organization working in tandem with country coordinators (who are generally FETP, PHSWOW, or FELTP program directors) in each country. Country coordinators are responsible for overall country level implementation and reporting to the secretariat. The secretariat reports to the board at least twice a year. In between board meetings, the secretariat works with established board subcommittees to implement the agreed upon plans and resolutions.

### Partnerships

Apart from the internal growth seen as a result of a larger membership (Figure 1), AFENET has recorded significant growth in external partnerships. Beginning with USAID, CDC, TEPHINET and WHO in 2004-2006, AFENET has since added at least 25 partners.

The Division Public Health Surveillance and Workforce Development (DPHSWD) of the Center of Global Health at CDC participated in the founding of AFENET. DPHSWD has brought in other CDC divisions including the Global AIDS Program (GAP) to support the mission of improving public health in Africa through field epidemiology capacity development. CDC has provided technical assistance in several areas for example, in developing training curricula, monitoring and evaluation, and in integrating new areas into FETPs and FELTPs (e.g. veterinary field epidemiology, non-communicable disease, and Expanded Program for Immunization (EPI)). Other partners include international non-profit organizations, universities in Europe and the US, Foundations, international networks, other US Federal Agencies (e.g., the National Institutes of Health), and the European Union.

Table 1 shows the grants awarded to AFENET from proposals submitted between 2006 and 2011. These exclude sub-contracts or sub-agreements which AFENET was not involved in developing, but was involved as an implementer after they were awarded (e.g., sub-contracts with the Research Triangle Institute (RTI) and CDC Foundation). 

**Table 1:** Grants written by the African Field Epidemiology Network (AFENET) and funded 2006-2011

Focus Area	Submitted	Funding Period	Funding Partner	AFENET Role
Applied Epidemiology Training	2006	5 years	CDC/Department for Health and Human Services (HHS)*	Lead
Immunization Training for Managers	2007	4 years	Merck Foundation	Sub-recipient
Cholera Surveillance	2009	3 years	Gates Foundation	Sub-recipient
Health Diplomacy Training Nigeria	2009	1 year	Office for Global Health Affairs (OGHA), HHS	Lead
Advanced Epidemiological Research Training	2010	4 Years	European Union	Sub-recipient
Laboratory Systems Strengthening	2010	5 years	CDC/HHS	Lead
Field Epidemiology Training	2010	5 years	CDC/HHS*	Lead
Influenza Surveillance	2010	16 months	WHO	Sub-recipient
Field Epidemiology training in Tanzania	2011	5 years	CDC Tanzania	Lead
Field Epidemiology training in Nigeria	2011	5 years	CDC Nigeria	Lead
Outbreak Investigation	2011	1 year	USAID-RESPOND	Sub-recipient

*: Substantial funding from USAID to AFENET received through a Cooperative Agreement

Since 2005, AFENET and its partners have been involved in a number of advocacy events including: meetings with various potential donors, and circulation of electronic and print materials. This has resulted in an augmented membership, partnerships, and a growing pool of trained epidemiologists and public health laboratorians. AFENET also holds biennial Scientific Conferences for all the FETPs and FELTP in the network. In 2010 AFENET co-hosted the Global FETP and FELTP meeting with TEPHINET in Cape Town, South Africa.

Table 2 shows the number of trainees and graduates from the 2-year programs in AFENET. Programs established after 2006 represent over 70% of current enrolment into FELTPs, FETPs, and PHSWOWs in Africa. Some of the programs receive a large portion of their funding through AFENET, while others are only partially supported by the network. 

**Table 2:** Number of epidemiologists trained through 2-year programs

Program	Established	Current trainees	Graduates
Zimbabwe	1993	43	143
Uganda	1994	30	225
Kenya	2004	29	63
South Africa	2007	23	10
Ghana	2007	15	11
Nigeria	2008	52	13
Tanzania	2008	23	11
Ethiopia	2009	38	13
Mozambique	2009	11	0
Central Africa (with Cameroon, Central African Republic, and the Democratic Republic of the Congo	2010	18	0
Rwanda	2010	15	0
West Africa (with Burkina Faso, Mali, Togo, and Niger)	2010	12	0
**Total**		**309**	**489**

### Challenges

AFENET has as its most immediate challenges; communication and language (there are three major official languages in AFENET countries English, French, and Portuguese), maintaining cohesion among members, and dealing with rapid growth. AFENET has addressed the language and communication challenge by drawing on a wide pool of expertise from graduates and FETP/FELTP faculty who speak the language and understand the culture of a particular country. AFENET has endeavored to engage early on all stakeholders when implementing programs in new countries in order to develop a common understanding of the mission to ensure that all activities and programs receive the commitment of all stakeholders. In a network such as AFENET, different members join with different expectations, and unless expectations are clarified from the outset, this can create room for tension. On the other hand, what members are expected to bring or contribute to the network must be made clear. As AFENET has grown, this area has not been given due attention. Over time, we have developed clear documentation that spells out the roles and responsibilities of the network to its members and vice versa. As the organization has grown, some members have felt excluded from certain activities, while some have felt over burdened. This is a challenge that needs to be addressed continuously, as a one-size-fits-all solution has not been found. AFENET has had to rapidly build its own internal capacity, as well as that of its members to coordinate and manage a growing portfolio of work.

## Discussion

We have described the origin and evolution of AFENET, an organization that has grown in geographic reach, scope and contribution to public health systems and workforce strengthening in Africa. The founding members and partners of AFENET came together in 2004 and 2005, and used their individual and collective strengths to establish the organization. AFENET and its membership have employed a competency-based approach to public health training for the various tiers of the continent's health systems. AFENET's creation was driven by the needs of its membership, countries in the region, as well as the public health workforce needs of Africa. This organization is owned by African institutions and continues to exist to serve the interests and needs of its membership and the continent.

The growth in FETPs and FELTPs recorded in the region has been a result of a deliberate effort by programs in the region working within a south-south framework, and with partners from the north to help countries establish these programs. This expansion was borne out of the vision set out by the founders of AFENET in 2004 and 2005. The geographical coverage of AFENET provides an important platform that local, regional and international players in the health sector could leverage to address key health priorities to meet regional and international targets. AFENET has established firm and robust linkages with ministries of health, universities and other health actors that are a foundation for successful health programs and interventions. The AFENET members include the 36 countries that WHO describes as being in human resources for health crisis, and AFENET can be used to address the public health workforce shortages in those countries [1].

Based on the AFENET experience a regional network can address the unique peculiarities in a region in an efficient and practical manner. However the field-based model of training utilized by AFENET member programs, though effective, is resource-intensive. The average cost of training one epidemiologist through the 2 year program offered by FELTPs is approximately $40,000. Some of the earlier programs (e.g., Uganda and Zimbabwe) have devised various mechanisms for sustainability beyond partner support such as strengthening country ownership (e.g., through advocacy with an emphasis on showcasing the impact of the programs in their respective countries, and reductions in the cost of training). In the short term, there is still need to mobilize external financial resources from donors to support existing FELTPs and FETPs and to develop new ones. AFENET and its members will need to continually innovate, and continue to build partnerships. Programs will need to look both within their countries, as well as externally for support. Internal support could come from trainees (e.g., by paying tuition), MOHs, the private sector and the country's development partners.

## Conclusion

AFENET was developed to address a clearly-recognized need within Africa to develop a network that would serve public health systems development, looking beyond the original founders, and using the existing capacity of the founders and partners to help other countries. Each partner brought their strengths to the table in helping form AFENET and set it on a path of growth and viability. The establishment of AFENET was impelled by the need for FETPs and FELTPs in Africa to work together in a systematic way to address the common and unique needs of Africa and the African programs. AFENET will need to continue to innovate, strengthen old alliances, and build new ones to continue fulfilling its mission and vision over the next decade and beyond, as part of the regional IDSR strategy and within the global IHR framework to strengthen surveillance and response core capacity development, as well as meeting other disease control and prevention priorities.  
